# Roles and Regulation of Voltage-gated Calcium Channels in Arrhythmias

**DOI:** 10.19102/icrm.2019.101006

**Published:** 2019-10-15

**Authors:** Jared Kushner, Xavier Ferrer, Steven O. Marx

**Affiliations:** ^1^Division of Cardiology, Department of Medicine, Vagelos College of Physicians and Surgeons, Columbia University, New York, NY, USA

**Keywords:** Arrhythmias, calcium, calcium channels, heart

## Abstract

Calcium flowing through voltage-dependent calcium channels into cardiomyocytes mediates excitation–contraction coupling, controls action-potential duration and automaticity in nodal cells, and regulates gene expression. Proper surface targeting and basal and hormonal regulation of calcium channels are vital for normal cardiac physiology. In this review, we discuss the roles of voltage-gated calcium channels in the heart and the mechanisms by which these channels are regulated by physiological signaling pathways in health and disease.

## Introduction

In the heart, calcium (Ca^2+^) entry through the voltage-gated Ca^2+^ channel initiates muscle excitation–contraction coupling. The influx of Ca^2+^ also contributes to the plateau phase of the action potential, pacemaker activity in nodal cells, and the modulation of critical cellular processes including metabolism and gene expression. Thus, a Ca^2+^ influx via voltage-gated Ca^2+^ channels in the heart links membrane depolarization to cellular functions. In this review, we will discuss mechanisms of Ca^2+^ handling in the heart and how dysfunctions of voltage-gated Ca^2+^ channels can lead to arrhythmias. Ca^2+^ channels are modulated by voltage, Ca^2+^, posttranslational modifications, and protein–protein interactions, which will also be reviewed. Finally, we will discuss existing pharmacological therapies that target voltage-gated Ca^2+^ channels.

### Structure and cellular electrophysiological function

Six classes of voltage-gated Ca^2+^ channels exist that can be classified by membrane voltage activation (low versus high), susceptibility to pharmacologic antagonists, and rate of inactivation **(Table 1)**; these include the T-, L-, N-, P-, Q-, and R-type channels. Of these, only long-lasting (L)- and transient (T)-type Ca^2+^ channels are expressed in cardiomyocytes.^1,2^ In Ca^2+^ channel nomenclature, the chemical symbol of calcium, Ca, is followed by a subscript “V,” denoting voltage as the primary regulator, and two numerical identifiers corresponding to the α_1_ subunit gene subfamily and the order of discovery within that subfamily, respectively.^3,4^ Ca_V_1.1, Ca_V_1.2, Ca_V_1.3, and Ca_V_1.4 exhibit relatively long-lasting currents and are referred to as L-type Ca^2+^ channels. Ca_V_3.1, Ca_V_3.2, and Ca_V_3.3, which are T-type Ca^2+^ channels, exhibit transient Ca^2+^ currents and are activated at more negative potentials relative to L-type Ca^2+^ channels **(Table 1)**.

**Figure 1:**
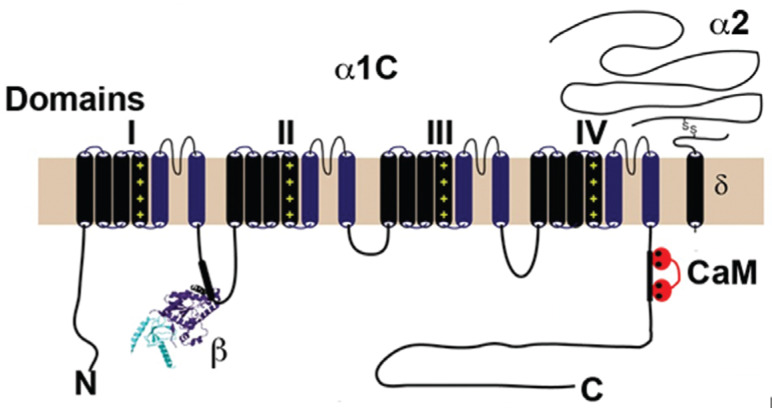
Schematic of cardiac α_1C_, β, and α_2_ subunit topology. The β subunit binds to the α-interaction domain in the I–II loop of the α_1C_ subunit. CaM binds to the C-terminus of α_1C_.

Voltage-gated Ca^2+^ channels are composed of the pore-forming α_1_ subunit and several auxiliary subunits including β and α_2_δ **(Figure 1)**. Four homologous domains, each with six transmembrane helices and a pore loop between S5 and S6 forming the α_1_ subunit. Alternating, positively charged arginine or lysine residues at every third or fourth position in S4 of each domain impart voltage sensitivity.^5^ The α_1_ subunit contains binding sites for most regulators and drugs, whereas the β, α_2_δ, and γ subunits contribute to trafficking, anchoring, and regulatory functions. The pore region contains binding sites for all major L-type Ca^2+^ channel-blocking agents including dihydropyridines, phenylalkylamines, and benzothiazepines **(Table 1)**.^6^

There are four β-subunit genes (Ca_V_β_1–4_). The β_2_ subunit is the predominantly expressed isoform in the adult heart. In all of the different β subunits, the guanylate kinase (GK) and Src-homology 3 (SH3) domains are very similar, whereas the N-termini (variable region 1), the linker between SH3 and GK (variable region 2), and the C-termini (variable region 3) are quite different.^7–9^ All β subunits interact with the pore-forming α subunit via the intracellular loop between transmembrane domains I and II **(Figure 1)**. In a cell-specific manner, β subunits can increase trafficking of the channel to the plasma membrane and modulate both activation and inactivation. In mice, global or cardiac-specific deletion of the *Cacnb2* gene leads to abnormal heart development and embryonic death.^10^ Conditional deletion of the β_2_ subunit in adult mouse cardiomyocytes causes a 96% or so reduction in β_2_ protein expression, but surprisingly only a 29% reduction in Ca^2+^ current, with no obvious cardiac impairment,^11^ implying that, in adult hearts, the β_2_ expression may be expendable. However, interpretation of this result is ambiguous, as it is complicated by the remnant (~4%) β_2_ expression as well as the presence of other Ca_V_β isoforms expressed in adult cardiomyocytes.^9^ Moreover, a contrasting viewpoint was provided by a study in which short hairpin RNA–mediated knockdown of β_2_ in adult rat myocytes substantially diminished Ca^2+^ current.^12^

To definitively address the controversies regarding the role of β subunits in mediating the trafficking and regulation of Ca^2+^ channels in the heart, we created transgenic mice lines with three mutations in the α-interaction domain in the I–II loop of the α_1C_ subunit, which renders the pore-forming α_1C_ subunit incapable of binding β subunits. With this new model, we definitively demonstrate in vivo that the β subunit binding to α_1C_ is not required for trafficking and that the basal function of β-less Ca^+2^ channels is only minimally altered.^13^

The α_2_δ subunit is a 175-kDa single transmembrane protein encoded by four genes (*Cacna2d1*, *Cacna2d2*, *Cacna2d3*, and *Cacna2d4*) with multiple splice variants. Although the messenger RNAs of α_2_δ 1 through 3^14^ have been identified in human myocardium, only α_2_δ-1 is known to bind with Ca_V_1.2 **(Figure 1)**.

Ca^2+^ channels are inactivated in both voltage- and Ca^2+^-dependent manners, but Ca^2+^-dependent inactivation is the dominant mechanism. L-type Ca^2+^ channels associate with calmodulin (CaM) **(Figure 1)**, which modulates both Ca^2+^-dependent inactivation and Ca^2+^-dependent facilitation, where Ca^2+^ currents increase after repetitive stimulation.^15^ The importance of CaM regulation of L-type Ca^2+^ channels in the heart has been demonstrated by overexpressing in adult cardiac myocytes a mutated CaM protein that cannot bind Ca^2+^, leading to very long action potentials because of the loss of Ca^2+^-dependent inactivation.^16^ Although Ca^2+^ entry via the Ca^2+^ channel can contribute to Ca^2+^-dependent inactivation, remarkably, it is specifically the Ca^2+^ released from the sarcoplasmic reticulum via the ryanodine receptors that is the primary determinant of Ca^2+^-dependent inactivation.

### Roles of L- and T-type calcium channels in the heart

**Figure 2:**
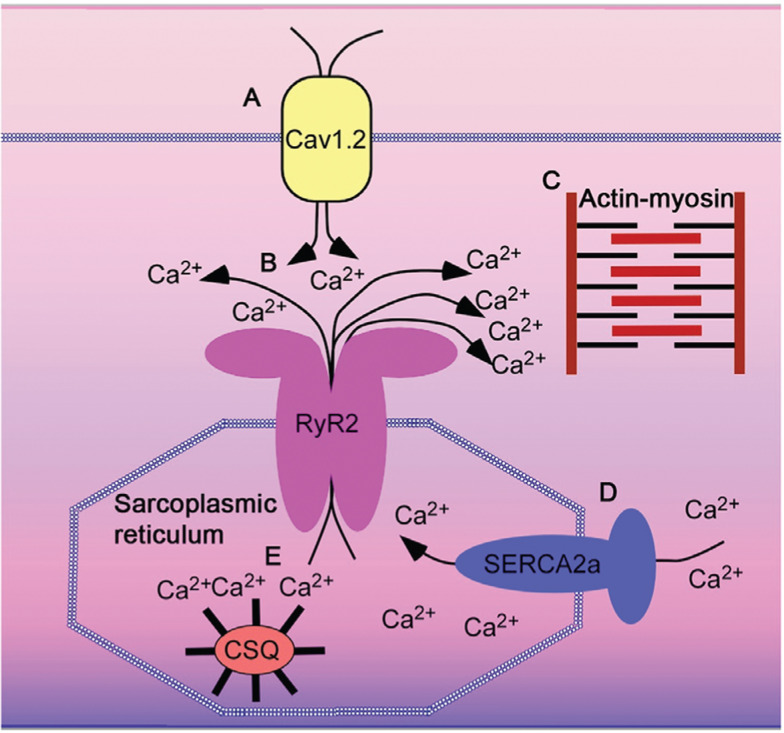
Excitation–contraction coupling in cardiomyocytes. **A and B:** Ca^2+^ entry via Ca_V_1.2 causes Ca^2+^-induced Ca^2+^ release via the ryanodine receptor (RyR2). **C:** Ca^2+^ binds to troponin C, inducing cross-bridging between actin and myosin. **D:** Ca^2+^ is pumped back into the sarcoplasmic reticulum via SERCA2a. The same amount of Ca^2+^ that enters the cell via Ca_V_1.2 is pumped out of the cells via the Na^+^–Ca^2+^-exchanger and Ca^2+^ pumps (not shown). **E**: Ca^2+^ binds to CSQ in the sarcoplasmic reticulum.

**Figure 3:**
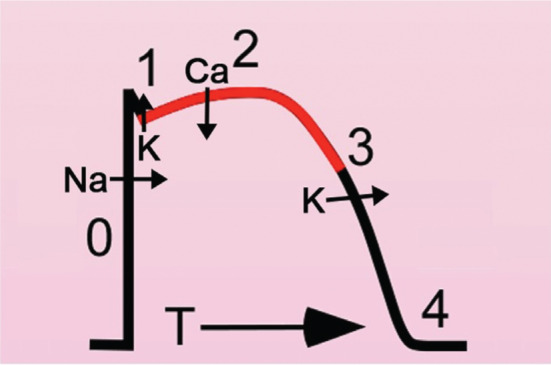
Diagram of cardiac action potential showing phases 0, 1, 2, 3, and 4. During phase 0, Na^+^ influx initiates the cardiac action potential. During phase 2, Ca^2+^ entry via the L-type Ca^2+^ channel initiates excitation–contraction coupling.

Understanding the regulation of myocyte Ca^2+^ regulation is essential to understanding cardiac arrhythmogenesis. Ca_V_1.2 is situated on the transverse tubules in close proximity with ryanodine receptors (RyR2), which are intracellular Ca^2+^ release channels located on the sarcoplasmic reticulum. Ca^2+^ entry through the L-type Ca^2+^ channels triggers ryanodine receptors to release Ca^2+^ from the sarcoplasmic reticulum into the cytoplasm as part of a process known as Ca^2+^-induced Ca^2+^ release **(Figure 2)**.^17^ Ca^2+^ influx via Ca^2+^-channel current and Ca^2+^ release via ryanodine receptors are required for myofilament activation **(Figure 2)**. Ca^2+^ binds to troponin C on the thin filament, allowing the myosin heads to bind to actin.^18^ The strength of contraction is proportional to the concentration of Ca^2+^ surrounding the myofilaments. In order to fully relax myocytes in preparation for the next heartbeat, the amount of Ca^2+^ that enters the cardiac cell during steady state must equal the amount of Ca^2+^ that leaves the cell.^18^ The reduction in cellular Ca^2+^ concentration is driven by Ca^2+^ transport via the sarcoplasmic reticulum Ca^2+^–adenosine triphosphate pump (SERCA) and the sarcolemmal Na^+^/Ca^2+^ exchanger **(Figure 2)**. During the action potential, voltage-gated Ca^2+^ channels open and allow Ca^2+^ to flow down its electrochemical gradient, causing the plateau phase (phase 2) of the action potential **(Figure 3)**. Outside of the t-tubules, L-type Ca^2+^ channels are also localized to caveolae—membrane invaginations important for concentrating proteins that are essential for the coordinating responses to extracellular signals—wherein Ca^2+^ influx can control signal transduction pathways. In ventricular myocytes, the Ca^2+^ current is mediated nearly entirely by the L-type Ca^2+^ channel, Ca_V_1.2. In atrial and especially pacemaker cells, the L-type Ca^2+^ channel isoform Ca_V_1.3, which activates at more negative potentials, contributes to the late phase 4 depolarization that underlies these cells’ automaticity **(Table 1)**.

In contrast to L-type Ca^2+^ current, T-type Ca^2+^ current has little effect on cardiomyocyte excitation–contraction coupling in the heart.^19^ The high density of T-type Ca^2+^ current in nodal cells^20^ and embryonic cardiomyocytes,^21^ however, is consistent with their putative role in pacemaker function. T-type Ca^2+^ channels contribute to triggered or pacemaker activity because they activate at even more negative potentials than L-type Ca^2+^ channels **(Table 1)**. However, they produce smaller peak Ca^2+^ currents and cannot substitute for L-type Ca^2+^ channels because T-type Ca^2+^ channels do not target to the sarcolemmal–sarcoplasmic reticulum junctions and therefore cannot initiate sarcoplasmic Ca^2+^ release.

### Pharmacology

There are three main chemical classes of organic Ca^2+^ channel drugs, specifically dihydropyridine (prototype: nifedipine), phenylalkylamines (prototype: verapamil), and benzothiazepines (prototype: diltiazem). All three classes of drugs bind within a single overlapping region close to the pore and the proposed activation gate.^22,23^ These drugs interfere with the voltage-dependent cycling of the channel.^24–26^ The uncharged dihydropyridines, which possess higher affinity for the inactivated channel conformation (voltage- or use-dependent block), induce and stabilize inactivated channel states.^24–27^ Smooth-muscle Ca_V_1.2 channels are more sensitive to inhibition by dihydropyridines than cardiac Ca_V_1.2 channels because the inactivated channel states are favored in arterial smooth muscle cells due to the relatively depolarized membrane potential of these cells and the splice variant of the S6 segment of domain 1, which is specifically expressed in this tissue.^25,28,29^

Ca_V_1.3 is less sensitive to dihydropyridines than Ca_V_1.2 is. Phenylalkylamines and benzothiazepines bind to the open and inactivated states with high affinity and stabilize the inactivated channel states, slowing recovery from inactivation, leading to use-dependent inhibition.^30,31^ Therefore, inhibition increases with higher heart rates, rationalizing the use of verapamil for tachyarrhythmias. Whereas verapamil and diltiazem always reduce inward Ca^2+^ currents, some dihydropyridines, such as (−)-BAY-K-8644 and (+)-SDS-202-791, are gating modifiers that act as agonists, increasing current amplitudes, tail currents, and single-channel open probability.^27^

### Disease states/channelopathies

Prolongation of the action-potential duration increases the loading of Ca^2+^ within the cells due to prolonged Ca^2+^ entry and a reduced diastolic interval for Ca^2+^ efflux. Moreover, some L-type Ca^2+^ channels become available again during the prolonged action-potential duration, and the channels reactivate, creating an inward Ca^2+^ influx, which can cause early afterdepolarizations.^32^ Increased L-type Ca^2+^ current also contributes to delayed afterdepolarizations and Ca^2+^-evoked arrhythmias, which occur after repolarization is complete and are exacerbated by sarcoplasmic reticulum Ca^2+^ overload. Mutations in L-type Ca^2+^ channels have been associated with inherited arrhythmia syndromes.

Timothy syndrome, a multisystem disorder characterized by a prolonged QT interval and syndactyly as well as variably penetrant phenotypes of autism spectrum disorders, craniofacial abnormalities, and hypoglycemia,^33^ is caused by the loss of voltage-dependent inactivation.^33^ The heterogeneous phenotype reflects the distribution of expression in the heart, brain, kidney, gastrointestinal tract, immune system, smooth muscle, testis, and pituitary and adrenal glands **(Table 1)**. The diagnosis is typically made within the first few days of life due to fetal bradycardia caused by functional 2:1 atrioventricular block. When completely expressed, Timothy syndrome is typically lethal within the first years of life. Repolarization is markedly prolonged in most patients with Timothy syndrome, with the corrected QT interval often exceeding 550 ms to 600 ms.^34^ Congenital cardiac defects are present in 60% of patients and cardiac hypertrophy and ventricular dilatation have been reported to occur in 50% of patients.^33–35^ Ventricular arrhythmias are the most frequent cause of death. There are no systematic studies assessing the best therapeutic strategy for patients with Timothy syndrome published to date. The available evidence supports the use of β-blockers and late Na^+^ channel blockers, and the use of implantable cardioverter-defibrillators for primary prevention is also reasonable.^36–39^ Close monitoring of glucose levels is essential, however, since β-blockers can mask the hypoglycemia caused by Timothy syndrome.

Mutations in 19 genes have been identified as associated with the Brugada phenotype, causing either a decrease in inward Na^+^ or Ca^2+^ currents or an increase in outward K^+^ currents.^40^ The resultant outward shift in the balance of currents active during phases 1 and 2 of the epicardial action potential allows for the already prominent transient outward K^+^ current to augment phase 1 repolarization. If the membrane potential is repolarized too much, L-type Ca^2+^ channels fail to activate, leading to a reduction in the action potential plateau predominantly in the right ventricular subepicardial cells in which the transient outward K^+^ current is most prominent. Loss-of-function mutations in the pore-forming α_1C_ subunit, the β_2b_ subunit, and the α_2_δ_1_ subunit have also been linked to Brugada, early repolarization, and short-QT syndromes.^41–44^ Agents that augment L-type Ca^2+^ currents, such as β-adrenergic agonists, have been shown to have therapeutic efficacy in Brugada syndrome.^40,45–47^

Short-QT syndrome^48^ is one of the rarest inheritable cardiac channelopathies, characterized by an accelerated cardiac repolarization. It is an autosomal-dominant disease with five identified causative genes, including three that encode for K^+^ channels (*KCNH2*, *KCNQ1*, and *KCNJ2)* and two that encode for subunits of the L-type Ca^2+^ channels (*CACNA1C* and *CACNB2*).^49–51^ Mutations in the Ca_V_1.2 genes *CACNA1C* and *CACNB2b* have also been associated with both idiopathic ventricular fibrillation and early repolarization syndrome.^43^

A mutation in *CACNA1D*, which encodes Ca_V_1.3, was identified in a Pakistani family with pronounced bradycardia resulting from nonconducting Ca_V_1.3 channels.^52^ A loss of Ca_V_1.3 reduces automaticity in pacemaker cells. Taken together, mutations of the core subunits of the L-type Ca^2+^ channels cause various cardiac syndromes and arrhythmias, including long-QT syndrome, Timothy syndrome, Brugada syndrome, short-QT syndrome, early repolarization, and bradycardia.

### Posttranslational regulation of calcium channels

Epinephrine and norepinephrine bind to β-adrenergic receptors in cardiomyocytes, the activation of which augments inotropy, lusitropy, and chronotropy.^18^ The activity of both protein kinase A (PKA) and Ca^2+^-CaM-activated protein kinase (CaMKII) increases with β-adrenergic stimulation, and both kinases provoke a rise in Ca_V_1.2 activity. The heightened activation of Ca_V_1.2, in turn, triggers increased contractility and Ca^2+^-responsive signaling pathways, which contribute to the pathogenesis of heart failure and hypertrophy.^53,54^

The molecular mechanism responsible for the β-adrenergic regulation of cardiac Ca_V_1.2 has remained a mystery. Experiments expressing recombinant Ca_V_1.2 in cultured cells (which have been, up until recently, the primary means of studying Ca_V_1.2 regulation) have not given a clear answer, since β-adrenergic regulation is not reliably reconstituted in standard cell lines and cardiomyocytes are irrevocably altered when cultured ex vivo.^55,56^ Thus, studies are required in native systems. The failure thus far to identify any single site as essential for β-adrenergic modulation led us to propose an alternative hypothesis: that a combination of phosphorylation sites in α_1C_ is required for β-adrenergic stimulation of Ca_V_1.2. Since β-adrenergic regulation of cardiac Ca_V_1.2 is conserved in vertebrates, we identified conserved PKA consensus sequences in the α_1C_ subunit of five species: mouse, rat, rabbit, guinea pig, and human. We then generated α_1C_ transgenic mice in which we replaced the 17 conserved consensus PKA phosphorylation sites that were not previously studied and the five conserved PKA/CaMKII phosphorylation sites that were known to be nonessential.^57–60^ Surprisingly, we found that none of these PKA consensus phosphorylation sites were necessary.^61^ Instead, we found that β*-*subunit binding to the Ca_V_1.2 α_1C_ subunit, but not PKA phosphorylation of β, is absolutely essential for the augmentation of Ca^2+^ current and cardiac contractile response to β-adrenergic−related PKA stimulation.^13^ These findings identify the key regulatory mechanisms impacting β-adrenergic regulation of Ca^2+^ influx and contractility in the heart.

Ca_V_1.2 is also a major target of CaMKII, and the resulting Ca^2+^-dependent facilitation of Ca_V_1.2 current, observed as a positive “staircase” of Ca^2+^ current in which current amplitude increases and inactivation slows over a series of repetitive pulses, is a powerful feed-forward effect on Ca^2+^ signaling in the heart. It is likely that phosphorylation of both α_1C_ and β_2_ subunits are required for CaMKII potentiation.^62,63^

## Conclusions

Ca^2+^ channels are absolutely essential regulators of intracellular Ca^2+^, automaticity, and contractility. The channels are regulated by a macromolecular complex consisting of core subunits and kinases, phosphatases, cytoskeletal proteins, and adaptor proteins. The dysfunction of the channels, caused by either genetic or acquired factors, is associated with heart failure, hypertrophy, or arrhythmias. Future ideal goals include providing a greater molecular understanding of the mechanisms underlying Ca^2+^ channel subcellular targeting, function, and modulation in cardiomyocytes in health and disease.

## Figures and Tables

**Table 1:** Properties of Voltage-gated Ca^2+^ Channels^64–68^

Isoform	Type	Gene	Localization	Antagonist	Activation Threshold
Ca_V_1.1	L	*CACNA1S*	Skeletal muscle	DHP, PLZ, BNZ	~ –20 mV
Ca_V_1.2	L	*CACNA1C*	Heart, nervous system, smooth muscle, adrenal gland, pancreas, kidney, cochlea	DHP, PLZ, BNZ	~ –20 mV
Ca_V_1.3	L	*CACNA1D*	Heart, nervous system, kidney, adrenal gland, pancreas, lung, testis, cochlea	DHP, PLZ, BNZ	~ –40 mV
Ca_V_1.4	L	*CACNA1F*	Retina	Unknown	~ –40 mV
Ca_V_2.1	P/Q	*CACNA1A*	Nervous system, smooth muscle, pancreas, cochlea	ω-agatoxin IVA	
Ca_V_2.2	N	*CACNA1B*	Nervous system, pancreas	ω-conotoxin GVIA	~ –35 mV
Ca_V_2.3	R	*CACNA1E*	Nervous system, heart, cochlea, pancreas, lung	SNX-482, Pb^2+^	~ –30 mV
Ca_V_3.1	T	*CACNA1G*	Heart, nervous system, pancreas, smooth muscle, kidney	Mibefradil, kurtoxin, Ni^2+^	~ –60 mV
Ca_V_3.2	T	*CACNA1H*	Heart, nervous system, smooth muscle, kidney	Mibefradil, kurtoxin, Ni^2+^	~ –60 mV
Ca_V_3.3	T	*CACNA1I*	Nervous system	Mibefradil, kurtoxin, Ni^2+^	~ –70 mV

BNZ: benzothiazepines; DHP: dihydropyridines; PLK: phenylalkylamines.
